# Author Correction: National parochialism is ubiquitous across 42 nations around the world

**DOI:** 10.1038/s41467-021-27677-8

**Published:** 2022-01-05

**Authors:** Angelo Romano, Matthias Sutter, James H. Liu, Toshio Yamagishi, Daniel Balliet

**Affiliations:** 1 Experimental Economics Group, Max Planck Institute for Research on Collective Goods, Bonn, Germany; 2 Leiden University, Social, Economic and Organizational Psychology, Leiden, The Netherlands; 3 Department of Economics, University of Cologne, Cologne, Germany; 4 Department of Public Finance, University of Innsbruck, Innsbruck, Austria; 5 School of Psychology, Massey University, Auckland, New Zealand; 6 Graduate School of International Corporate Straegy, Hitotsubashi University, Tokyo, Japan; 7 Department of Experimental and Applied Psychology, Vrije Universiteit Amsterdam, Amsterdam, The Netherlands; 8 Institute of Brain and Behavior (IBBA), Amsterdam, The Netherlands

Correction to: *Nature Communications* 10.1038/s41467-021-24787-1, published online 22 July 2021.

The original version of the Source Data associated with this Article included an error in the sheet for Supplementary Figure 1, which contained two incorrect data points. The correct content of cell C15 is ‘2.0048’ instead of the original, incorrect ‘2.1224’ and the correct content of cell D37 is ‘4.5090’ instead of the original, incorrect ‘4.4090’. The HTML has been updated to include a corrected version of the Source Data.

## Supplementary information

Updated Source Data

