# Supplementary material for: National parochialism is ubiquitous across 42 nations around the world
Source: Nat Commun. 2021 Jul 22;12:4456. doi: 10.1038/s41467-021-24787-1 (PMC8298626; doi:10.1038/s41467-021-24787-1)
Supplement: Supplementary file 1 — Supplementary Information [file 41467_2021_24787_MOESM1_ESM.pdf]

Supplementary Information for

“National Parochialism is Ubiquitous Across 42 Nations around the World”

Angelo Romano, Matthias Sutter, James Liu, Toshio Yamagishi, Daniel Balliet

Correspondence to: a.romano@fsw.leidenuniv.nl

1. **Supplementary Notes**
   1.1. Model results
      1.1.1. Full main model
      1.1.2. Controls and robustness checks
      1.1.3. Gender and education
      1.1.4. Payment model
      1.1.5. Expectations model
      1.1.6. Cross-national models
      1.1.7. Model with cultural clusters
      1.1.8. Ingroup favoritism vs outgroup derogation
      1.1.9. Meta-analytic approach on national parochialism across nations
      1.1.10. Cleveland plots of ingroup vs outgroup and ingroup vs strangers
   1.2. Cross-cultural indicators
   1.3. Cultural distance: analytic approach
   1.4. R-codes
   1.5. Instructions

2. **Supplementary References**

1. Supplementary Notes

1.1. Model results

In this section, we provide the full report of the results of the models. First, we run the model with only the Contrast 1 and public vs private variables (Supplementary Table 1), then we add models with controls and attention checks (Supplementary Table 2 and 3) and models about the interaction of gender and education with Contrast 1 (Supplementary Table 4 and 5). We also include models of the nations where we used incentives (Supplementary Table 6), models with expectations as an outcome variable (Supplementary Table 7), models that tested cross-cultural predictions on national parochialism and cooperation (Supplementary Table 8 and 9), a model on cultural clusters predicting national parochialism (Supplementary Table 10), and a model that tests whether we observe outgroup derogation (Supplementary Table 11).

1.1.1. Full main model

In this section, we report the model with the main treatments and their interaction predicting cooperation: Contrast 1 (Ingroup = 1, Outgroup and Stranger = 0), Observability (public choice = 1, private choice = 0). Results show that people are more cooperative with ingroup compared to outgroup and stranger (national parochialism), and that people cooperate more when their choice is public, compared to when their choice is private. The interaction between the two treatments is not significant.

Supplementary Table 1. Mixed-effect model of Contrast 1 and Observability predicting cooperation.

Cooperation	*b*	*SE*	*t*	*p*
Contrast1	0.29	0.02	13.17	<.001
Observability	0.12	0.008	14.50	<.001
Contrast1×Observability	-0.007	0.01	-0.54	0.59
$N_{\text{observations}}$	220,132*			
N_{subjects}	18,411			

Note. Contrast1 = ingroup vs outgroup + stranger treatments; Observability = public vs private treatments; × = interaction term. *total observations are slightly less than the expected number of

decisions (12) multiplied by the number of subjects (18,411) due to some missing cases in Japan (in Japan, due to a randomization error participants were randomly allocated to fewer decisions). The table shows estimates without adjustments for multiple comparisons. All tests were two-sided. Source data are provided as a Source Data file.

1.1.2. Controls and robustness checks

In this section, we report two models. In Supplementary Table 2, we report a model with the main treatments and controls: Contrast 1 (Ingroup = 1, Outgroup and Stranger = 0), Observability (public choice = 1, private choice = 0), Quick Understanding (1 = participant responded correctly to the comprehension question in the first attempt, 0 = participant did not respond correctly to the comprehension question in the first attempt), Age (continuous variable of exact year), Gender (1 = Man, 0 = Woman), Education (ordinal variable where 1 = elementary school, 2 = middle school, 3 = high school, 4 = some college, 5 = bachelor degree, 6 = graduate school or higher).

Supplementary Table 2. Mixed-effect model of Contrast 1 and Observability predicting cooperation, including controls and quick understanding proxy.

Cooperation	*b*	*SE*	*t*	*p*
Contrast 1	0.29	0.02	13.28	<.001
Observability	0.12	0.008	14.67	<.001
Contrast1×Observability	-0.007	0.01	-0.52	0.60
Quick Understanding	0.006	0.03	0.17	0.86
Age	0.005	0.001	3.615	<.001
Gender (Male = 1)	0.18	0.03	5.473	<.001
Education	0.06	0.015	3.62	<.001
$N_{observations}$	217,484			
$N_{subjects}$	18,189			

Note. Contrast1 = ingroup vs outgroup + stranger treatments; Observability = public vs private treatments; Quick Understanding = people who provided the correct answer in their first attempt + others); × = interaction term. The table shows estimates without adjustments for multiple comparisons. All tests were two-sided. Source data are provided as a Source Data file.

Results show that controlling for quick understanding, age, gender and education, people are still more cooperative with ingroup compared to outgroup members and stranger (national parochialism), and that people cooperate more when their choice is public, compared to when

their choice is private. The interaction between the two treatments also remain not significant. We also find that men, compared to women, cooperate more with strangers, that age has a positive relation with cooperation and that more educated people cooperate more compared to less educated people.

In Supplementary Table 3, we report a model with the main treatments and controlling for people who did not pass the attention check: Contrast 1 (Ingroup = 1, Outgroup and Stranger = 0), Observability (public choice = 1, private choice = 0), Attention check (1 = participant passed the attention check, 0 = participant did not pass the attention check). Results show that controlling for the attention check people are still more cooperative with ingroup compared to outgroup and stranger (national parochialism), and that people cooperate more when their choice is public, compared to when their choice is private. The interaction between the two treatments also remain not significant. People that passed the attention check cooperated less with strangers compared to people that did not pass the attention check.

Supplementary Table 3. Mixed-effect model of Contrast 1 and Observability predicting cooperation, including people that failed the attention check (*N* = 27,527).

Cooperation	*b*	*SE*	*t*	*p*
Contrast 1	0.25	0.02	12.43	<.001
Observability	0.10	0.007	14.01	<.001
Contrast1×Observability	-0.01	0.01	-0.92	0.35
Attention check	-0.17	0.01	-6.15	<.001
$N_{observations}$	328,855			
$N_{subjects}$	27,527			

Note. Contrast1 = ingroup vs outgroup + stranger treatments; Observability = public vs private treatments; Attention check = people who passed the attention check + people who did not pass the attention check); × = interaction term. The table shows estimates without adjustments for multiple comparisons. All tests were two-sided. Source data are provided as a Source Data file.

1.1.3. Gender and education

In this section, we report two models. In Supplementary Table 4, we report a model with the main treatments and controls: Contrast 1 (Ingroup = 1, Outgroup and Stranger = 0),

Observability (public choice = 1, private choice = 0), Quick Understanding (1 = participant responded correctly to the comprehension question in the first attempt, 0 = participant did not respond correctly to the comprehension question in the first attempt), Age (continuous variable of exact year), Gender (1 = Man, 0 = Woman), and the interaction between Gender and Contrast 1.

Results show that controlling for quick understanding, age, and gender, people are still more cooperative with ingroup compared to outgroup members and stranger (national parochialism), and that people cooperate more when their choice is public, compared to when their choice is private. We also find that men, compared to women, cooperate more with strangers, and that age has a positive relation with cooperation. Importantly, we find an interaction between Gender and Contrast 1, suggesting that national parochialism is stronger among men, compared to women.

Supplementary Table 4. Mixed-effect model of Contrast 1 and Observability predicting cooperation, including the interaction between gender and national parochialism.

Cooperation	*b*	*SE*	*t*	*p*
Contrast 1	0.26	0.009	26.62	<.001
Observability	0.12	0.006	17.60	<.001
Contrast1×Male	0.03	0.01	2.04	0.04
Quick Understanding	0.02	0.03	0.58	0.56
Male	0.17	0.03	5.015	<.001
Age	0.005	0.001	3.82	<.001
$N_{\text{observations}}$	217,484			
N_{subjects}	18,189			

Note. Contrast1 = ingroup vs outgroup + stranger treatments; Observability = public vs private treatments; Quick Understanding = people who provided the correct answer in their first attempt + others); × = interaction term. The table shows estimates without adjustments for multiple comparisons. All tests were two-sided. Source data are provided as a Source Data file.

In Supplementary Table 5, we report a model with the main treatments and controls: Contrast 1 (Ingroup = 1, Outgroup and Stranger = 0), Observability (public choice = 1, private choice = 0), Quick Understanding (1 = participant responded correctly to the comprehension

question in the first attempt, 0 = participant did not respond correctly to the comprehension question in the first attempt), Age (continuous variable of exact year), Education (ordinal variable where 1 = elementary school, 2 = middle school, 3 = high school, 4 = some college, 5 = bachelor degree, 6 = graduate school or higher), and the interaction between Education and Contrast 1.

Results show that controlling for quick understanding, age, and education, people are still more cooperative with ingroup compared to outgroup members and stranger (national parochialism), and that people cooperate more when their choice is public, compared to when their choice is private. We also find that higher educated people are more cooperative than lower educated people. Importantly, we find an interaction between Education and Contrast 1, suggesting that national parochialism is stronger among lower educated people, compared to higher educated people.

Supplementary Table 5. Mixed-effect model of Contrast 1 and Observability predicting cooperation, including the interaction between education and national parochialism.

Cooperation	*b*	*SE*	*t*	*p*
Contrast 1	0.34	0.03	11.99	<.001
Observability	0.12	0.007	17.57	<.001
Contrast1×Education	-0.02	0.006	-2.335	0.02
Quick Understanding	0.008	0.03	0.23	0.82
Age	0.006	0.001	4.132	<.001
Education	0.06	0.015	3.72	<.001
$N_{observations}$	217,484			
$N_{subjects}$	18,189			

Note. Contrast1 = ingroup vs outgroup + stranger treatments; Observability = public vs private treatments; Quick Understanding = people who provided the correct answer in their first attempt + others); × = interaction term. The table shows estimates without adjustments for multiple comparisons. All tests were two-sided. Source data are provided as a Source Data file.

1.1.4. Payment model

In this section, we report the model with the main treatments and their interaction with the use of incentives in Brazil, India and Poland: Contrast 1 (Ingroup = 1, Outgroup and Stranger

= 0), Observability (public choice = 1, private choice = 0), Payment (Incentives = 1, Hypothetical scenario = 0). Results show that interactions between Contrast 1 and Payment, and between Contrast 1 and Observability are not significant. This suggests that the use of incentives does not change national parochialism levels and cooperation under public vs private situations. There is also no significant main effect of Payment on cooperation, although as expected, if any, people in the incentive treatment cooperate less compared to people in the hypothetical scenario. As incentives are hypothesized to decrease experimental demand effects[1], these findings provide support for the idea that our study design which relies on hypothetical decisions was not contaminated with demand characteristics. This result, together with past research that found no differences across between-subjects and within-subjects designs in the extent of national parochialism and observability[2,3,4], makes it very unlikely, in our view, that demand characteristics might constitute a potential confound.

Supplementary Table 6. Mixed-effect model of Contrast 1 and Observability predicting cooperation, controlling for the use of incentives vs hypothetical situations in Brazil (*N* = 832) , India (*N* = 834) and Poland (*N* = 776).

Cooperation Payment Nations (Brazil, India, Poland)	*b*	*SE*	*t*	*p*
Contrast 1	0.15	0.04	3.73	0.02
Observability	0.07	0.008	2.70	0.01
Payment	-0.14	0.026	-1.57	0.12
Contrast 1 × Payment	0.01	0.039	0.33	0.74
Observability × Payment	0.04	0.037	0.96	0.34
$N_{observations}$	29,316			
$N_{subjects}$	2,443			

Note. Contrast1 = ingroup vs outgroup + stranger treatments; Observability = public vs private treatments; Payment = real stakes + hypothetical choices); × = interaction term. The table shows estimates without adjustments for multiple comparisons. All tests were two-sided. Source data are provided as a Source Data file.

1.1.5. Expectations model

In this section, we report the model with the main treatments, their interaction, and controls predicting expected partner's cooperation: Contrast 1 (Ingroup = 1, Outgroup and Stranger = 0), Observability (public choice = 1, private choice = 0), Quick Understanding (1 = participant responded correctly to the comprehension question in the first attempt, 0 = participant did not respond correctly to the comprehension question in the first attempt), Age (continuous variable of exact year), Gender (1 = Man, 0 = Woman), Education (ordinal variable where 1 = elementary school, 2 = middle school, 3 = high school, 4 = some college, 5 = bachelor degree, 6 = graduate school or higher).

Supplementary Table 7. Mixed-effect model of Contrast 1 and Observability predicting expectations about partner's cooperation.

Expectations	*b*	*SE*	*t*	*p*
Contrast 1	0.10	0.03	3.45	.001
Observability	0.07	0.008	7.80	<.001
Contrast1×Observability	0.02	0.01	1.45	0.15
Quick Understanding	-0.44	0.03	14.083	<.001
Age	0.008	0.001	6.153	<.001
Gender	0.13	0.03	4.21	<.001
Education	0.02	0.01	1.45	0.15
$N_{observations}$	217,544			
$N_{subjects}$	18,194			

Note. Contrast1 = ingroup vs outgroup + stranger treatments; Observability = public vs private treatments; Quick Understanding = people who responded correctly in their first attempt + people who did not respond correctly in their first attempt); × = interaction term. The table shows estimates without adjustments for multiple comparisons. All tests were two-sided. Source data are provided as a Source Data file.

Results show that people expect more cooperation when their partner is an ingroup member compared to when the partner is an outgroup member or a stranger, and that people expect more cooperation more when the choice is public, compared to when their choice is private. The interaction between the two treatments is not significant. Men expect more cooperation from their partners, compared to women. Older participants expected more

cooperation from others. People that provided the correct answer in their first attempt in the comprehension check expected less cooperation from their partners, and there is no difference in expectations among people with different education levels.

1.1.6. Cross-national models

In this section, we report the results of independent mixed effects models (with nations as random intercepts) where each cross-national indicator predicts national parochialism (Supplementary Table 8) or cooperation (Supplementary Table 9). The outcome variable of Supplementary Table 8 is the output of a principal component analysis that assumes that the data across the 12 decisions load on two factors (ingroup vs outgroup + stranger treatments). This can be interpreted as the influence of partner's nationality on cooperation (national parochialism). In Supplementary Table 8, higher scores for each of the cross-national indicators indicate higher rule of law, government effectiveness, religiosity, church attendance, prevalence of diseases, and relational mobility. Results show that none of the cross-national indicators predict national parochialism.

Supplementary Table 8. Independent models of pre-registered nation level indicators predicting national parochialism.

National parochialism	*b*	*SE*	*t*	*p*	$N_{nations}$	$N_{observations}$
Rule of law	-0.02	0.03	-0.58	0.56	42	18,411
Government effectiveness	0.009	0.03	0.28	0.77	42	18,411
Religiosity	0.027	0.03	0.78	0.44	37	16,395
Church attendance	0.055	0.03	1.62	0.11	37	16,395
Prevalence of diseases	-0.03	0.03	-0.79	0.43	42	18,411
Relational mobility	0.02	0.04	0.45	0.66	26	10,995

Note. See section 1.2 for details about the sources of the cross-cultural indices. All indicators were standardized. Please also note that even if here we report the indicators of the preregistered hypotheses, none of the other cross-national indicators predicted national parochialism. The table shows estimates without adjustments for multiple comparisons. All tests were two-sided. Source data are provided as a Source Data file.

The outcome variable of Supplementary Table 9 is the output of a principal component analysis that assumes that the data across the 12 decisions load on one factor. This can be interpreted as the cooperativeness of one person independent of the treatments (general level of cooperation). Regarding the results, the interpretation of the sign of the estimate is intuitive for most of the indicators. We list here the ones for which interpretation is counter-intuitive. Higher scores in confidence to institutions means lower confidence in institutions. Higher scores in Indulgence vs Restraint mean higher indulgent nations, while higher scores in Individualism vs Collectivism mean higher individualistic nations. Higher scores in self-expression (petition) mean lower endorsement of petitions.

Supplementary Table 9. Independent mixed models of nation-level indicators predicting general levels of individual cooperation.

Overall cooperation	*b*	*SE*	*t*	*p*	N_{nations}	$N_{observations}$
Environmental Threats						
Prevalence of infectitious diseases	-0.65	0.17	-3.90	<.001	42	18,411
Death to communicable disease	-0.46	0.24	-1.94	0.06	40	17,629
Economic indicators						
GDP per capita	0.23	0.19	1.16	0.25	41	18,019
Adjusted national income	0.28	0.19	1.45	0.15	40	17,629
Human development index	0.22	0.20	1.13	0.27	41	18,019
Gini	-0.06	0.20	-0.28	0.78	35	15,633
Hofstede cultural dimensions						
Individualism vs collectivism	0.39	0.18	2.24	0.03	41	18,019
Uncertainty avoidance	-0.14	0.21	-0.67	0.51	37	16,459
Power distance	-0.49	0.17	-2.91	0.006	41	18,019
Long term orientation	-0.41	0.17	-2.38	0.023	39	17,239
Masculinity	-0.23	0.19	-1.24	0.22	41	18,019
Indulgence vs restraint	0.49	0.17	2.84	0.007	39	17,239
Preferences						
Patience	0.32	0.19	1.70	0.10	35	15,664
Trust	-0.08	0.20	-0.41	0.69	37	16,395
Risk	0.17	0.19	0.90	0.37	35	15,664
Positive reciprocity	0.06	0.20	0.31	0.76	35	15,664
Negative reciprocity	-0.06	0.20	-0.31	0.76	35	15,664
Altruism	-0.16	0.20	-0.80	0.43	35	15,664
Religion						
Religiosity	0.17	0.20	0.87	0.39	37	16,395
Church attendance	0.31	0.19	1.59	0.12	37	16,395
Belief in hell	0.28	0.20	1.38	0.18	35	15,286
Belief in heaven	0.19	0.25	0.75	0.46	27	11,505
Exposure to western church	0.58	0.19	3.11	0.003	36	16,005
% Christians	0.58	0.18	3.30	0.002	42	18,411
Social ecology						
Relational mobility	0.64	0.22	2.86	0.009	26	10,955
Net migration	0.508	0.19	2.69	0.011	41	18,019
Institutions						
Globalization	0.17	0.20	0.83	0.41	40	17,639
Cultural looseness (vs tighteness)	0.40	0.21	1.94	0.06	29	12,790
Press freedom	-0.48	0.18	-2.71	0.009	42	18,411
Intellectual autonomy	0.40	0.19	2.07	0.045	39	17,135

Civil liberties	0.30	0.19	1.60	0.12	42	18,411
Government effectiviness	0.06	0.19	0.31	0.76	42	18,411
Hierarchical values	-0.43	0.20	-2.12	0.04	38	16,855
Rule of law	0.28	0.19	1.48	0.15	42	18,411
Confidence in institutions						
Confidence in… the courts	0.09	0.21	0.46	0.65	29	12,911
…armed forces	0.30	0.20	1.54	0.13	37	16,395
…justice system	-0.23	0.29	-0.80	0.44	17	7,924
…the police	0.06	0.19	0.30	0.77	37	16,395
…the parliament	0.48	0.19	2.52	0.016	37	16,395
…the legal system	0.62	0.21	2.96	0.008	25	11,100
…the government	0.45	0.18	2.43	0.021	37	16,395
Self-expression values						
Self-expression (abortion)	0.44	0.19	2.36	0.024	37	16,395
Self-expression (homosexuality)	0.43	0.19	2.26	0.03	37	16,395
Self expression (petition)	-0.52	0.18	-2.94	0.006	37	16,395
Self expression (divorce)	0.56	0.18	3.08	0.004	37	16,395
Self expression (men job)	0.65	0.17	3.91	<.001	37	16,395

Note. See section 1.2 for details about the sources of the cross-cultural indices. All indicators were standardized. The table shows estimates without adjustments for multiple comparisons. All tests were two-sided. Source data are provided as a Source Data file.

1.1.7. Model with cultural clusters

In this section, we report independent mixed models split by cultural clusters with the main treatments predicting cooperation: Contrast 1 (Ingroup = 1, Outgroup and Stranger = 0), Observability (public choice = 1, private choice = 0). We clustered nations based on previous seminal work by Inglehart and Baker[5] which plot and categorize nations according to two dimensions: "traditional vs secular values" and "survival vs self-expression values". Moreover, similar to previous research[6], for Arabic speaking and Southern European nations we referred to Hofstede[7]. List of cultural clusters include: *Protestant* (Germany, Sweden, Netherlands, Finland), *English speaking* (UK, Canada, New Zealand, Australia, United States), *Confucian* (South Korea, Taiwan, Hong Kong, Japan, China, Singapore), *Orthodox* (Russia, Serbia), *Catholic Europe* (Italy, Spain, Portugal, Poland, Hungary), *Southern Europe* (Greece, Turkey),

South Asia (India, Pakistan, Philippines, Malaysia, Indonesia), *Latin America* (Argentina, Mexico, Brazil, Peru, Colombia, Venezuela, Panama, Bolivia), *Africa* (South Africa, Nigeria, Kenya), *Arabic speaking* (Egypt, Morocco). Those mixed models have participant ID as a level-2 random factor. Results show that people are more cooperative with ingroup compared to outgroup and strangers (national parochialism), and that people cooperate more when their choice is public, compared to when their choice is private. These results are found in all cultural clusters.

Supplementary Table 10. Independent mixed-models of observability and national parochialism predicting cooperation by cultural clusters.

Cooperation	*b*	*SE*	*t*	*p*
Protestant (Germany, Sweden, Netherlands, Finland, N_{obs}= 21,912)				
Contrast 1	0.30	0.02	14.78	<.001
Observability	0.13	0.02	6.91	<.001
English speaking (UK, Canada, New Zealand, Australia, United States, N_{obs}= 23,604)				
Contrast 1	0.36	0.02	17.82	<.001
Observability	0.09	0.02	4.70	<.001
Confucian (South Korea, Taiwan, Hong Kong, Japan, China, Singapore, N_{obs}= 27,184)				
Contrast 1	0.27	0.02	14.23	<.001
Observability	0.15	0.02	8.53	<.001
Orthodox (Russia, Serbia, N_{obs}= 9,324)				
Contrast 1	0.38	0.03	10.98	<.001
Observability	0.13	0.03	4.10	<.001
Catholic Europe (Italy, Spain, Portugal, Poland, Hungary, N_{obs}= 37,404)				
Contrast 1	0.29	0.02	17.35	<.001
Observability	0.13	0.01	8.45	<.001
Southern Europe (Greece, Turkey, N_{obs}= 9,744)				
Contrast 1	0.51	0.04	13.84	<.001
Observability	0.07	0.03	2.18	.02
South Asia (India, Pakistan, Philippines, Malaysia, Indonesia, N_{obs}= 28,776)				
Contrast 1	0.24	0.02	11.57	<.001
Observability	0.08	0.02	3.91	<.001
Latin America (Argentina, Mexico, Brazil, Peru, Colombia, Venezuela, Panama, Bolivia, N_{obs}= 38,508)				
Contrast 1	0.18	0.02	9.66	<.001
Observability	0.12	0.02	6.97	<.001
Africa (South Africa, Nigeria, Kenya, N_{obs}= 14,016)				
Contrast 1	0.16	0.03	4.92	<.001
Observability	0.13	0.03	4.23	<.001
Arabic speaking (Egypt, Morocco, N_{obs}= 9,660)				
Contrast 1	0.32	0.04	8.94	<.001
Observability	0.12	0.03	3.52	<.001

Note. Contrast1 = ingroup vs outgroup + stranger treatments; Observability = public vs private treatments; × = interaction term. The table shows estimates without adjustments for multiple comparisons. All tests were two-sided. Source data are provided as a Source Data file.

1.1.8. Ingroup favoritism vs outgroup derogation

In this section, we report the model with Contrast 2 (Outgroup vs Stranger) and its interaction with Observability predicting cooperation: Contrast 2 (Stranger = 1, Outgroup = 0), Observability (Public choice = 1, Private choice = 0). Results show that people are more cooperative with outgroup members compared to strangers. These findings (together with the findings of Supplementary Table 1) suggest that national parochialism seems to be motivated to benefit ingroup members (ingroup favoritism) instead of harming outgroup members (outgroup derogation). Moreover, the model show that people cooperate more when their choice is public, compared to when their choice is private. The interaction between the two treatments is not significant.

Supplementary Table 11. Mixed-effect model of Contrast 2 (outgroup vs stranger) and Observability predicting cooperation.

Cooperation	*b*	*SE*	*t*	*p*
Contrast2	-0.15	0.02	-8.25	<.001
Observability	0.12	0.01	10.74	<.001
Contrast2×Observability	-0.002	0.01	-0.16	0.87
$N_{\text{observations}}$	146,736			
N_{subjects}	18,411			

Note. Contrast2 = outgroup vs stranger; Observability = public vs private treatments; × = interaction term. The table shows estimates without adjustments for multiple comparisons. All tests were two-sided. Source data are provided as a Source Data file.

Moreover, we also tested whether identification with the own nationality was positively associated with national parochialism across nations. To do so, we run a mixed effect model of national identification predicting national parochialism score. National identification consists of a 1-item measure in which participants were asked to what extent they agreed or disagreed to the following statement: “I identify with my nationality” (1= totally disagree, 7 = totally agree). Supporting the idea that a motivation of ingroup favoritism drives national parochialism, we

found that national identification was positively associated with the national parochialism score ($b = 0.10$, $p < .001$).

1.1.9. Meta-analytic approach on national parochialism across nations

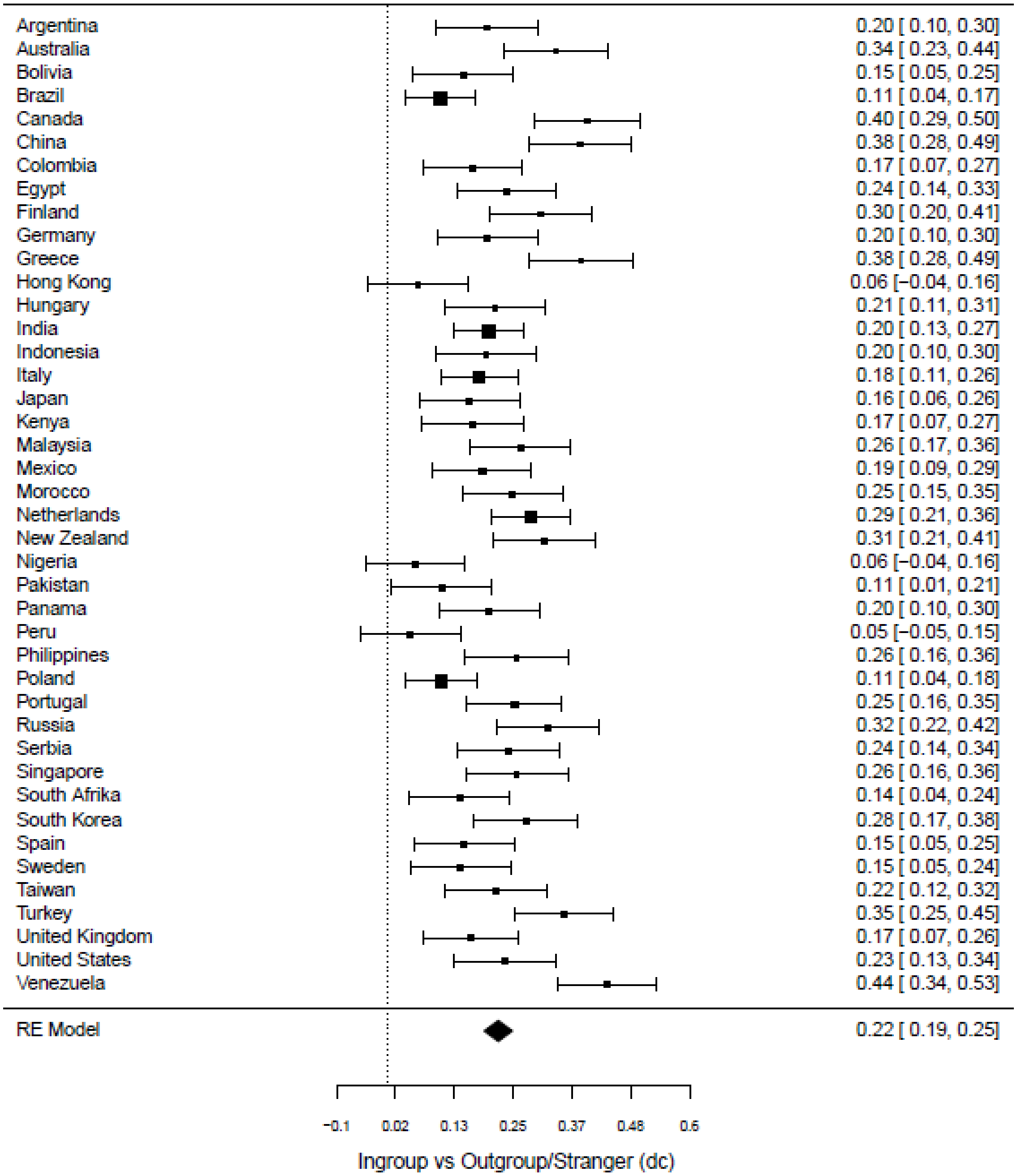

Supplementary Figure 1. Forest plot of national parochialism across nations. Forest plot displaying effect size of national parochialism predicting cooperation. For each nation, we report estimated effect size (Cohen's *d*) in each nation represented by a black square and 95% confidence interval. The size of the black square represents the sample size in each nation. The overall estimated population effect sizes are represented by the size of the black diamonds, which correspond to the 95% confidence intervals. Source data are provided as a Source Data file.

1.1.10. Cleveland plots of ingroup vs outgroup and ingroup vs strangers

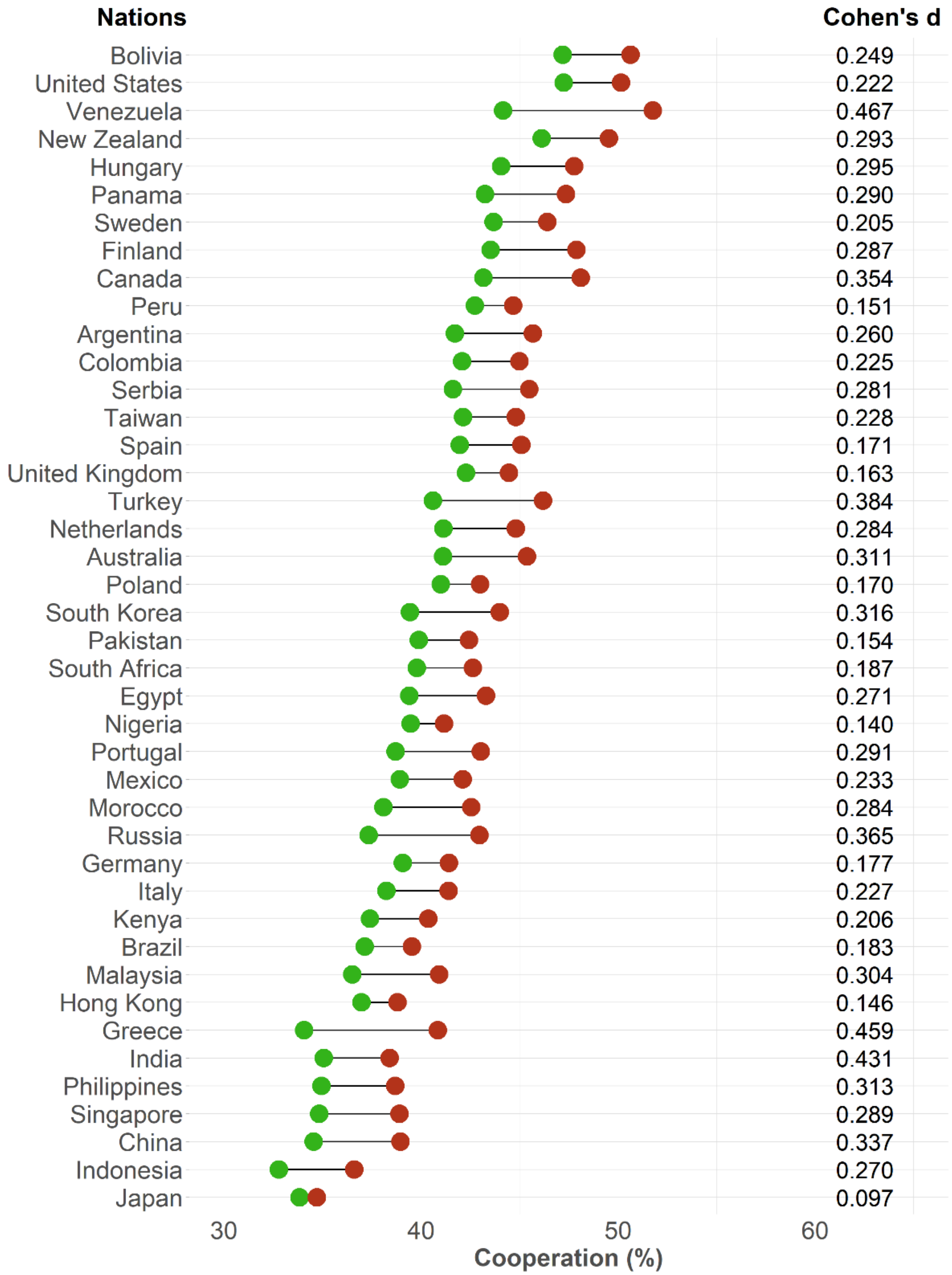

Supplementary Figure 2. Cleveland plot ingroup vs stranger. Cleveland dot plot showing the mean of cooperation (in percentage) with ingroup members (red dots) compared to unidentified strangers (green dots) across all the 12 decisions (including both the public and private treatments). Nations are sorted based on their average cooperation levels. The right-side column reports the estimated standardized mean difference (Cohen's d_c) of national parochialism (ingroup vs stranger) predicting cooperation. Source data are provided as a Source Data file.

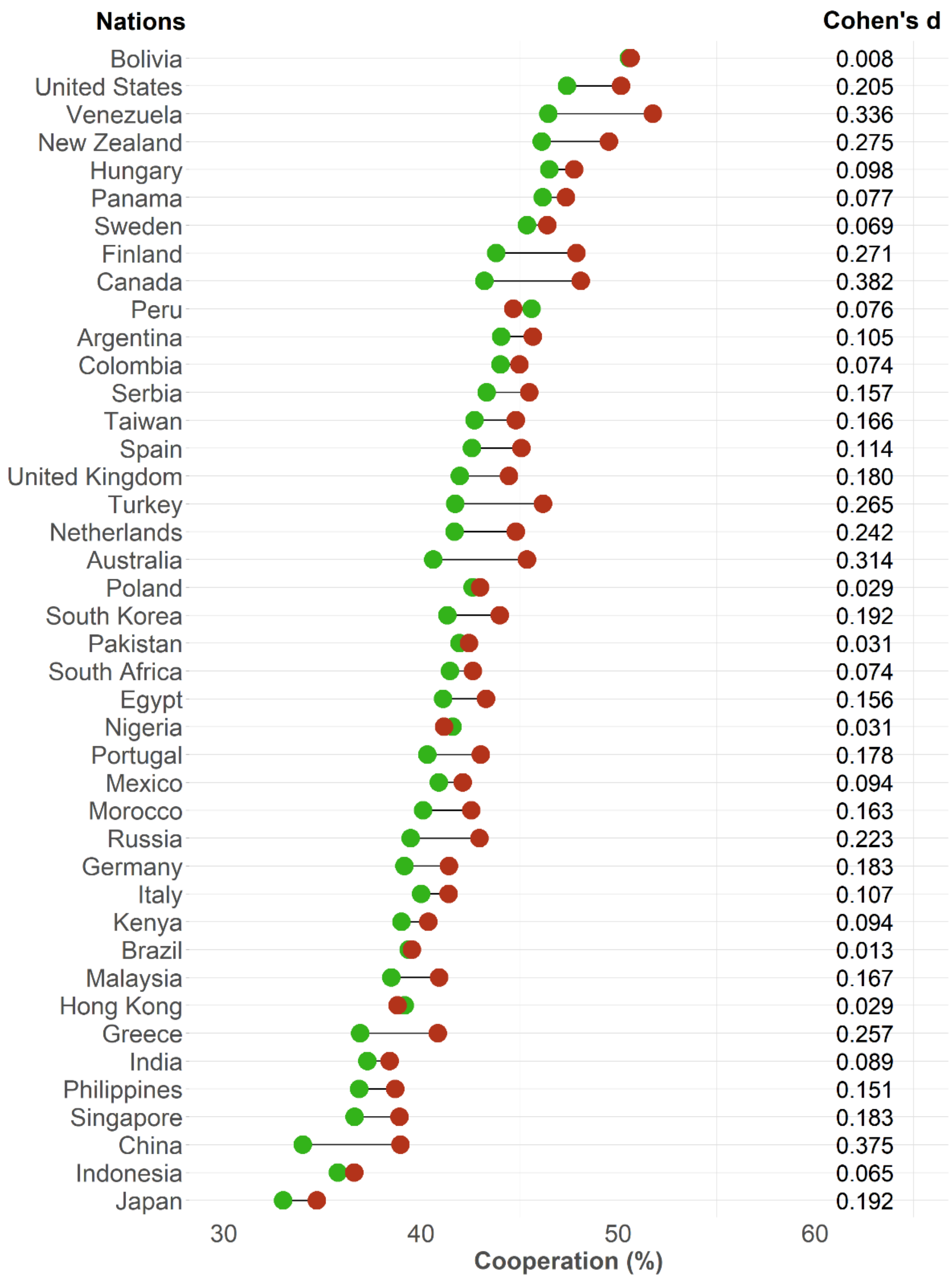

Supplementary Figure 3. Cleveland plot ingroup vs outgroup. Cleveland dot plot showing the mean of cooperation (in percentage) with ingroup members (red dots) compared to outgroup members (green dots) across all the 12 decisions (including both the public and private treatments). Nations are sorted based on their average cooperation levels. The right-side column reports the estimated standardized mean difference (Cohen's d_c) of national parochialism (ingroup vs outgroup) predicting cooperation. Source data are provided as a Source Data file.

1.2. Cross-cultural indicators

We considered several cross-national indicators. For all of them we retrieved the most recent available data from datasets, such as the World Bank, world values survey, Hofstede etc.

Supplementary Table 12. Summary of the cross-national indicators, their source, and year of measurement.

Indicators	Source	Year
Historical prevalence of pathogens	Murray, D. R., & Schaller, M. (2010)[8]	2010
Years of life lost to communicable disease	World Health Organization	2016
Relational mobility	http://relationalmobility.org/	2018
GDP per capita	World Bank	2017
Gini coefficient	World Bank	2017
Government effectiveness	World Bank	2017
Religiosity	World Value Survey (WVS)	1981-2014
Religious attendance	WVS	1981-2014
Belief in heaven	WVS	1981-2014
Belief in hell	WVS	1981-2014
Western Church Exposure	Schulz, Jonathan, Duman Bahrami-Rad, Jonathan Beauchamp (2018)[9]	2018
Confidence in the Armed Forces	WVS	1981-2014
Confidence in the Justice System	WVS	1981-2014
Confidence in the Legal System	WVS	1981-2014
Confidence in the Government	WVS	1981-2014
Confidence in the Courts	WVS	1981-2014
Confidence in the Parliament	WVS	1981-2014
Trust	Global Preference Survey (GPS)[10]	2016
Patience	Global Preference Survey (GPS)[10]	2016
Risk	Global Preference Survey (GPS)[10]	2016
Positive Reciprocity	Global Preference Survey (GPS)[10]	2016
Negative Reciprocity	Global Preference Survey (GPS)[10]	2016
Altruism	Global Preference Survey (GPS)[10]	2016
Individualism-Collectivism	https://www.hofstede-insights.com/product/compare-countries/	2010

Uncertainty avoidance	https://www.hofstede-insights.com/product/compare-countries/	2010
Power distance	https://www.hofstede-insights.com/product/compare-countries/	2010
Long-term orientation	https://www.hofstede-insights.com/product/compare-countries/	2010
Masculinity	https://www.hofstede-insights.com/product/compare-countries/	2010
Indulgence vs restraint	https://www.hofstede-insights.com/product/compare-countries/	2010
Survival vs. Self-Expression 1 (abortion)	WVS	1981-2014
Survival vs. Self-Expression 2 (homosexuality)	ESS	2002-2016
Survival vs. Self-Expression 3 (petition)	WVS	1981-2014
Survival vs. Self-Expression 4 (divorce)	WVS	1981-2014
Survival vs. Self-Expression 5 (men job)	ESS	2002-2016
Civil liberty	Freedom house	2018
Intellectual autonomy	Schwartz, S. H., & Boehnke, K. (2004)[11]	2004
Hierarchy	Schwartz, S. H., & Boehnke, K. (2004)[11]	2004
Human Development Index	http://hdr.undp.org/en/content/human-development-index-hdi	2016
Net Migration	World Bank	
Looseness vs tightness	Uz I. (2015)[12]	2015
Rule of law	Freedom house	2018

1.3. Cultural distance: analytic approach

We analyzed whether a measure of cultural distance between two nations predicted differences in national parochialism and cooperation. Cultural distance is a measure of the overall cultural differences between nations. To build this index, we retrieved bilateral cultural distance data from http://culturaldistance.muth.io/[13]. Cultural distance is calculated from data on beliefs, values and behaviors that people have about their own nation retrieved from the world value survey (two waves: 2005-2009; 2010-2014; for a complete report of the beliefs, values and behaviors used to calculate this indicator see[13]).

The world value survey dataset is composed by 170,247 participants from 80 nations (which altogether cover 85% of the world population). We used the bilateral distances in a simple regression as an IV to predict absolute differences (between pair of nations) of national parochialism and cooperation. We found cultural distance data in 36 out of the 42 nations (missing nations are Bolivia, Greece, Kenya, Panama, Portugal and Venezuela) investigated in our study. This gives a total of 630 unique bilateral cultural distance scores. Results show that bilateral cultural distances do not predict bilateral differences in national parochialism (b = -0.022, SE = 0.058, t-$value$ = -0.375, p = .701, R^2 = .001), but they predict bilateral differences in cooperation (b = 0.590, SE = 0.163, t-$value$ = 3.632, p < .001, R^2 = .021).

1.4. R-codes

Code of the main models.

*M0 <- lmer(Cooperation~Contrast_1*Observability + (Contrast_1|Nation) + (1|id), data=gdatac)*

*M1 <- lmer(Cooperation~Contrast_1*Observability + compr_check + age + Gender + Education + (Contrast_1|Nation) + (1|id), data=gdatac)*

*M2 <- lmer(Expectations~Contrast_1*Observability + compr_check + age + Gender + Education + (Contrast_1|Nation) + (1|id), data=gdatac)*

*M1_pay <- lmer(Cooperation~Contrast_1*Informed_PAYMENT + Observability*Informed_PAYMENT + (Contrast_1|Nation) + (1|id), data=gdatac)*

*M1__full <- lmer(Cooperation~Contrast_1*Observability + Valid_cases + (Contrast_1|Nation) + (1|id), data=Tdata)*

Code of the cross-cultural analysis. First, we selected two components (PC1 = overall cooperation; PC2= ingroup vs outgroup and strangers), then we ran mixed effect models where cross-national indicators predicted overall cooperation or national parochialism.

components<-prcomp(~ A1.COOP.INGR.OBS + A1.COOP.b + A3.COOP.OUT.OBS + A3.COOP.b + A5.COOP.STR.OBS + A5.COOP.b + A2.COOP.INGR.UNI + A2.COOP.b + A4.COOP.OUT.UNI + A4.COOP.b + A6.COOP.STR.UNI + A6.COOP.b, data = notresc, na.action = na.exclude)

components$x

summary(components)

notresc$COOPERATION <- components$x[,1]
notresc$NATIONAL PAROCHIALISM <- components$x[,2]

Cross-cultural models predicting cooperation.

```
m1 <- lmer(COOPERATION~ Prevalence_of_infectious_diseases_9_s   + (1|Nation) , data=final)
m2 <- lmer(COOPERATION~ Relational_mobility_Raw_s   + (1|Nation) , data=final)
m3 <- lmer(COOPERATION~ Power_distance_indexhofstede_s   + (1|Nation) , data=final)
m4 <- lmer(COOPERATION~ Individualism_vs_collectivism_s   + (1|Nation) , data=final)
m5 <- lmer(COOPERATION~ Indulgence_vs_restraint_s  + (1|Nation) , data=final)
m6 <- lmer(COOPERATION~ Globalization_s +     (1|Nation) , data=final)
```

```
m7 <- lmer(COOPERATION~ Percent_christian_s   + (1|Nation) , data=final)
m8 <- lmer(COOPERATION~ cult_tight_s  + (1|Nation) , data=final)
m9 <- lmer(COOPERATION~ Intellectual_Autonomy_s    + (1|Nation) , data=final)
m10 <- lmer(COOPERATION~ Uncertainty_Avoidance_s    + (1|Nation) , data=final)
m11 <- lmer(COOPERATION~ Tightness_score_s   + (1|Nation) , data=final)
m12 <- lmer(COOPERATION~ Deaths_to_communicable_disease_s    + (1|Nation) ,
data=final)
m13 <- lmer(COOPERATION~ Press_freedom_s  + (1|Nation) , data=final)
m14 <- lmer(COOPERATION~ Adjusted_net_national_income_s    + (1|Nation) , data=final)
m15 <- lmer(COOPERATION~ Human_Development_Index_s   + (1|Nation) , data=final)
m16 <- lmer(COOPERATION~ hierarchy_s    + (1|Nation) , data=final)
m17 <- lmer(COOPERATION~ patience_s    + (1|Nation) , data=final)
m18 <- lmer(COOPERATION~ vuln_disasters_s    + (1|Nation) , data=final)
m19 <- lmer(COOPERATION~ ChurchExpWest_s   + (1|Nation) , data=final)
m20 <- lmer(COOPERATION~ rule_of_law_FH_s  + (1|Nation) , data=final)
m21 <- lmer(COOPERATION~ net_migration_s  + (1|Nation) , data=final)
m22 <- lmer(COOPERATION~ ROL_together  + (1|Nation) , data=final)
m23 <- lmer(COOPERATION~GDP_per_capita_current_US_dollar_s + (1|Nation) ,
data=final)
m24 <- lmer(COOPERATION~ Active_memb_avg_s +(1|Nation) , data=final)
m25 <- lmer(COOPERATION~ avg_trust_s +(1|Nation) , data=final)
m26 <- lmer(COOPERATION~ avg_petition_s +(1|Nation) , data=final)
m27 <- lmer(COOPERATION~ avg_importance_god_s +(1|Nation) , data=final)
m28 <- lmer(COOPERATION~ avg_men_jobs_s +(1|Nation) , data=final)
m29 <- lmer(COOPERATION~ avg_divorce_s +(1|Nation) , data=final)
m30 <- lmer(COOPERATION~ avg_homosexual_s +(1|Nation) , data=final)
m31 <- lmer(COOPERATION~ avg_importance_religion_s +(1|Nation) , data=final)
m32 <- lmer(COOPERATION~ avg_conf_police_s +(1|Nation) , data=final)
m33 <- lmer(COOPERATION~ avg_conf_parliament_s +(1|Nation) , data=final)
m34 <- lmer(COOPERATION~ avg_conf_legal_system_s +(1|Nation) , data=final)
m35 <- lmer(COOPERATION~ avg_conf_government_s +(1|Nation) , data=final)
m36 <- lmer(COOPERATION~ avg_conf_armed_forces_s +(1|Nation) , data=final)
m37 <- lmer(COOPERATION~ avg_conf_justice_system_s +(1|Nation) , data=final)
m38 <- lmer(COOPERATION~ avg_conf_courts_s +(1|Nation) , data=final)
m39 <- lmer(COOPERATION~ avg_belief_hell_s +(1|Nation) , data=final)
m40 <- lmer(COOPERATION~ avg_belief_heaven_s +(1|Nation) , data=final)
m41 <- lmer(COOPERATION~ avg_abortion_s +(1|Nation) , data=final)
```

Preregistered cross-cultural models predicting national parochialism.

```
m1 <- lmer(NATIONAL PAROCHIALISM ~ Prevalence_of_infectious_diseases_9_s   +
(1|Nation) , data=final)
m2 <- lmer(NATIONAL PAROCHIALISM ~ Relational_mobility_Raw_s   + (1|Nation) ,
data=final)
m3 <- lmer(NATIONAL PAROCHIALISM ~ Government effectiveness   + (1|Nation) ,
data=final)
```

```
m4 <- lmer(NATIONAL PAROCHIALISM ~ Religiosity   + (1|Nation) , data=final)
m5 <- lmer(NATIONAL PAROCHIALISM ~ Church attendance   + (1|Nation) , data=final)
m6 <- lmer(NATIONAL PAROCHIALISM ~ Rule of law   + (1|Nation) , data=final)
```

1.5. Instructions

In this section, we include the instructions of the experiment. Regarding the decisions, we include three example of decisions (1 = partner is ingroup, public choice, 2 = partner is outgroup, public choice, 3 = partner is stranger, private choice). All the other decisions are combination of information provided in these 3 examples.

Information Sheet
Introduction. The study is being conducted by Professor ### and Professor ###

We aim at testing some theories about decision making. **For this reason, we kindly ask you to answer the survey seriously.**

Procedures. The purpose of this research is to examine decision making in different situations. **You** will interact with some other participants in some decision making tasks. **Then,** you will be asked to answer some questions about the decision making tasks. We estimate it will take no more than ***25 minutes*** to complete the study.

Risks/Discomforts. There are no anticipated risks for participating in this study.

Benefits. A potential benefit of participating is that you might learn something about decision-making that you might not have been aware of before. You may also be assigned to make a decision involving or being affected by someone from another nation.
Anonymity. All of your answers will be anonymous. Any information you provide will be stored indefinitely on the encrypted and password protected site, and on password-protected computers only. When presenting the results of this research, we will in no way focus on individual participants' responses and will instead present the findings in summary form. You will not be asked for information that would enable to identify you personally.

Compensation.
Independent Variable:
[no - payment condition]
You are playing for Monetary Units, a fictional currency that gauges how well you are doing at the decision-making task. These Monetary Units are meaningful in the context of the experiment, but have no value in the real world.
[payment condition]
Depending on you and others' decisions in the decision-making tasks, you will have **an opportunity to earn up to ## (*the currency and amount differed by society, but each amount was equal to 2.5 minutes average wage in that society).***

Participation & Rights. Your participation in this study is completely voluntary. You are free to

choose to withdraw from the study at any point.

Questions about the Research. If you would like to receive a summary of the results of this study, or have any questions, please email ##### at ####. This project has been reviewed and approved by #############. If you have any privacy or ethics concerns, please email ############################.

If you understand the information above and agree to participate in this research project, please click “I Agree” to start with this study. If you do not wish to participate right now, please close your web browser. Thank you for considering participating.

Welcome to the study. This is a study about decision making. The study involves **participants from many countries around the world**.

You will be asked to make decisions in several decision making tasks. You will be paired with a different person in each decision making task.

14. Before reading the instructions, please create a nickname for yourself (any combination of two letters and two numbers, such as x2f4)

Please read the instructions carefully because you have the possibility to **earn [Money]/[Monetary Units] [IV] based on your decisions and others' decisions.**

[Please treat each Monetary Unit (MU) as though it was worth 1.16 USD when making decisions.]/ [Each Monetary Unit (MU) is worth ## *(the currency and amount differed by society, but each amount was equal to 2.5 minutes average wage in that society)* when making decisions.] [IV]

(page break)

<u>Instructions</u>. In this task, you are paired with another person: PERSON B. Each of you receives 10 monetary units (MU).

[Each monetary unit is $$ cents. This amount is based on the average amount of pay for 2.5 minutes of work in your country.
When you interact with a person from a different country, then the value of an MU will be based on the average amount of pay for 2.5 minutes of work in that country. So, an MU has the same value across all the countries. **] IV FOR PAYMENT CONDITION ONLY**

You and PERSON B have the opportunity to send between 0 and 10 MU to the other and keep the remaining for yourself (in this example you send 2, and your partner sends 4).

Each MU that you and your partner send to the other will be doubled. For example, if you send 2 MU to PERSON B, PERSON B gets 4 MU. If PERSON B sends 4 MU to you, you get 8 MU.

Your final outcome is the result of what you keep for your self, and what you get from PERSON B multiplied by 2 (16 in this example).

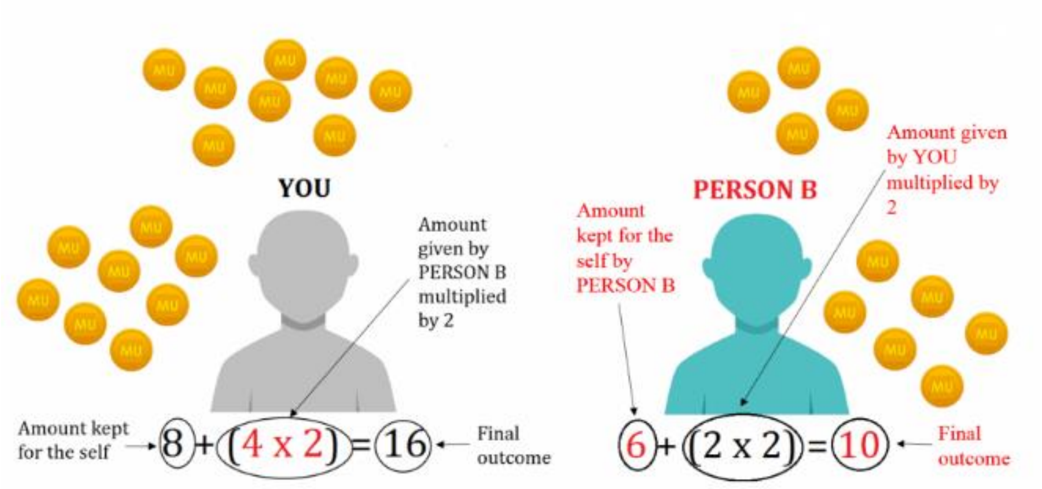

(page break)

In these tasks, you and other people from around the world will make several decisions.

Each decision will be made with a different partner.

(page break)

To make sure you have understood the instructions, please answer the following questions:

Remember: Both you and Person B begin the task with 10 MU

You send 4 MU. PERSON B sends 3 MU back to you. Then, (choose one)

YOU earn 12, PERSON B earns 10
YOU earn 15, PERSON B earns 12
YOU earn 12, PERSON B earns 15
YOU earn 4, PERSON B earns 3

Example *ingroup* and *public* treatment decision

In this round you will make a decision in the following situation

YOU

PERSON B: is from Italy

PLEASE NOTE: Your and your partner's decision in this round will be public. All participants will be given a link to see the results of your individual contribution under your nickname in a widely distributed blog: http://www.what-did-people-do.com

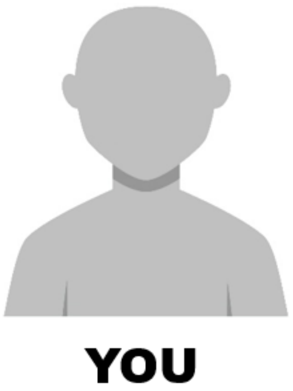

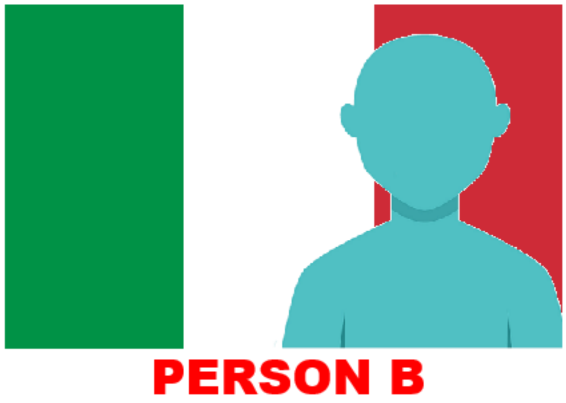

Click here if you are ready to make your decision

(page break)

Please make a decision in the following situation

YOU: NICKNAME HERE
PERSON B: is from Italy

PLEASE NOTE: Your and your partner's decision in this round will be public. All participants will be given a link to see the results of your individual contribution under your nickname in a widely distributed blog: http://www.what-did-people-do.com

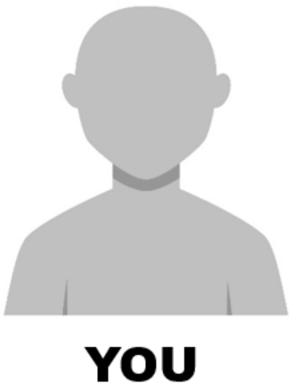

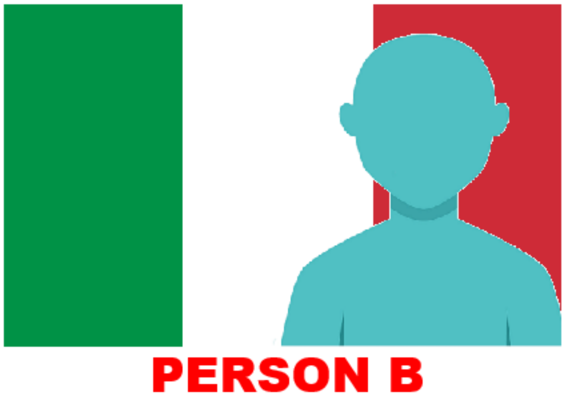

Number of MU you send to the PERSON B (click one number)

[Please treat each Monetary Unit (MU) as though it was worth 1.16 USD for decision-making purposes] IV FOR NO PAYMENT CONDITION ONLY
[Each Monetary Unit (MU) is worth ….] IV FOR NO PAYMENT CONDITION ONLY

(page break)

What do you expect from PERSON B?

YOU: NICKNAME HERE
PERSON B: is from Italy
PLEASE NOTE: Your and your partner's decision in this round will be public. All participants will be given a link to see the results of your individual contribution under your nickname in a widely distributed blog: http://www.what-did-people-do.com

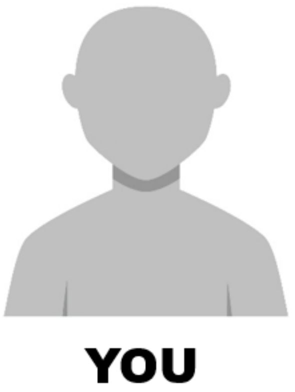

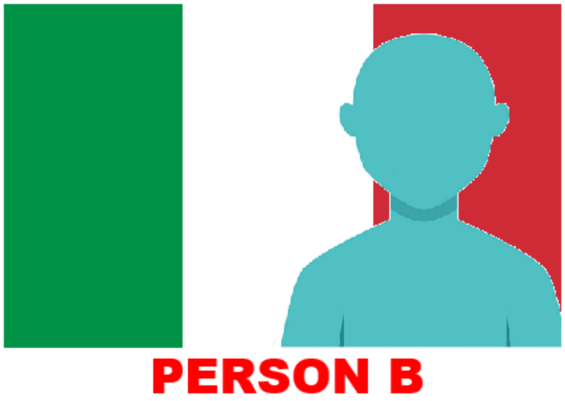

The number of MU you expect PERSON B will send to you

(before they get doubled)
Set the button to a number

(page break)

—

PLEASE NOTE: in the next page, you will make a decision with a different partner

—

(page break)

Example *outgroup* and *public* treatment decision

In this round you will make a decision in the following situation

YOU: NICKNAME HERE
PERSON B: is from one of 8 countries excluding Italy (Australia, Colombia, Germany, India, Nigeria, Serbia, Singapore, United States)
PLEASE NOTE: Your and your partner's decision in this round will be public. All participants will be given a link to see the results of your individual contribution under your nickname in a widely distributed blog: http://www.what-did-people-do.com

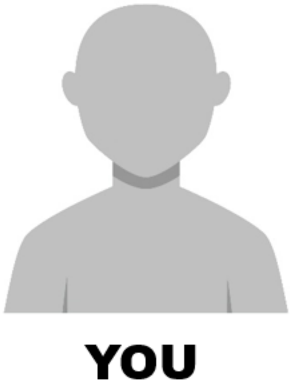

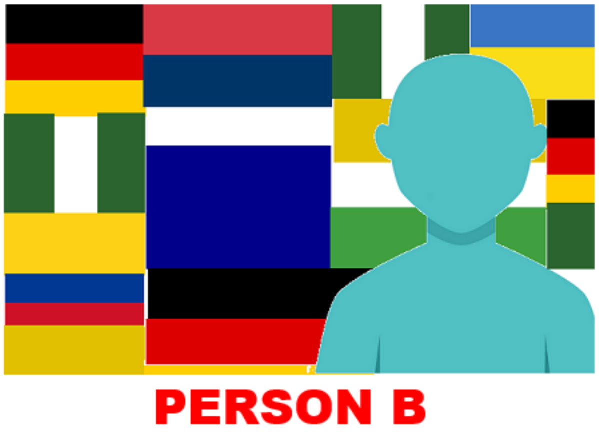

Please make a decision in the following situation

YOU: NICKNAME HERE
PERSON B: is from one of 8 countries excluding Italy (Australia, Colombia, Germany, India, Nigeria, Serbia, Singapore, United States)
PLEASE NOTE: Your and your partner's decision in this round will be public. All participants will be given a link to see the results of your individual contribution under your nickname in a widely distributed blog: http://www.what-did-people-do.com

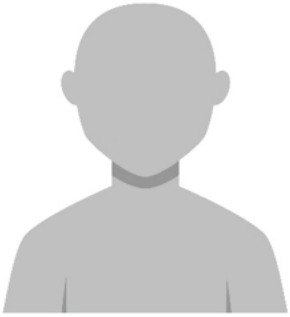

YOU

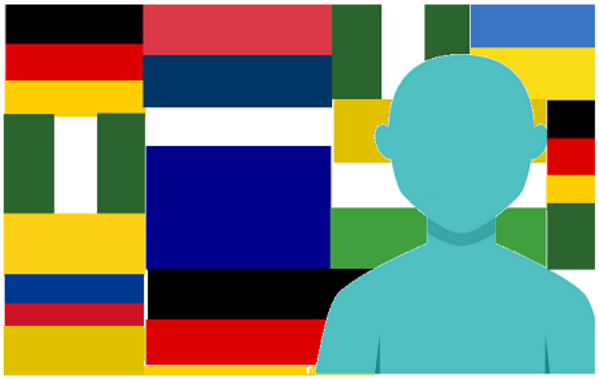

PERSON B

(page break)

What do you expect from PERSON B?

YOU: NICKNAME HERE
PERSON B: is from one of 8 countries excluding Italy (Australia, Colombia, Germany, India, Nigeria, Serbia, Singapore, United States)
PLEASE NOTE: Your and your partner's decision in this round will be public. All participants will be given a link to see the results of your individual contribution under your nickname in a widely distributed blog: http://www.what-did-people-do.com

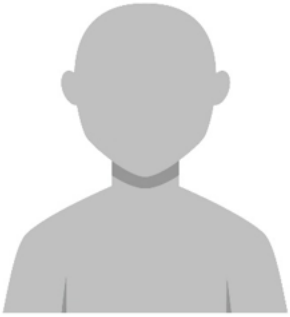

YOU

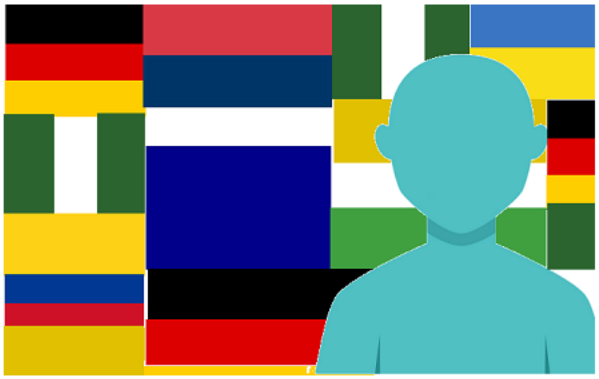

PERSON B

(page break)

—

PLEASE NOTE: in the next page, you will make a decision with a different partner

—

(page break)

Example *stranger* and *private* treatment decision

In this round you will make a decision in the following situation

YOU: unknown
PERSON B: unknown
PLEASE NOTE: Your and your partner's decision in this round will be private and not reported to other people

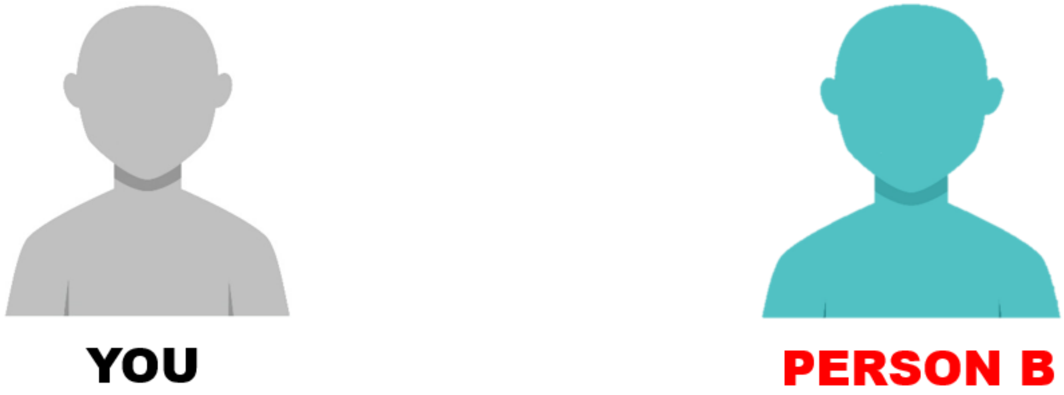

--

Please make a decision in the following situation

YOU: unknown
PERSON B: unknown
PLEASE NOTE: Your and your partner's decision in this round will be private and not reported to other people

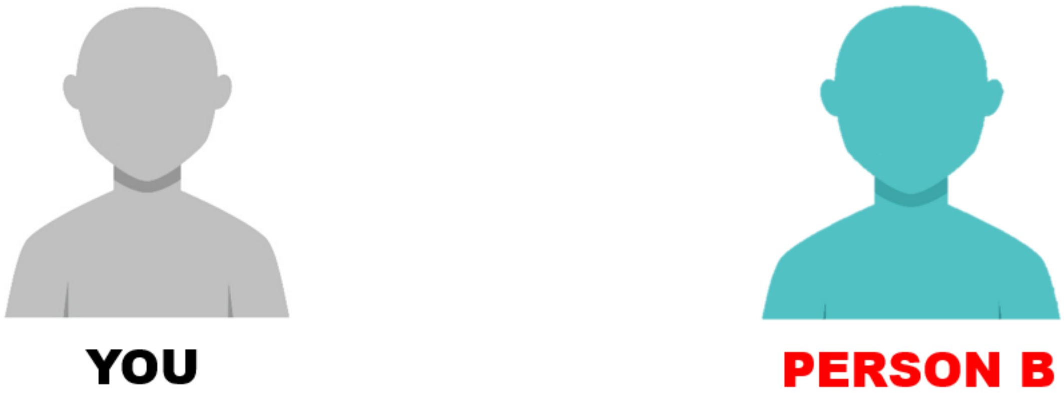

(page break)

What do you expect from PERSON B?

YOU: unknown
PERSON B: unknown
PLEASE NOTE: Your and your partner’s decision in this round will be private and not reported to other people

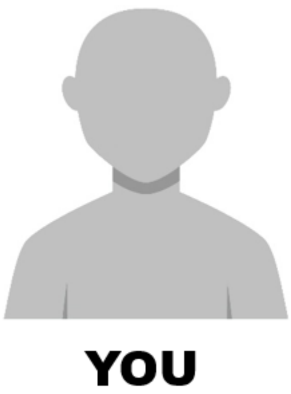

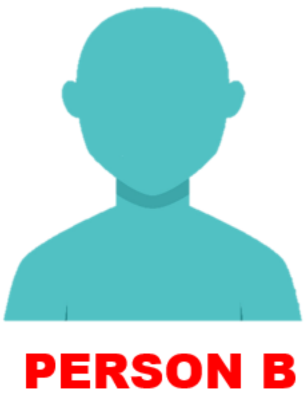

(page break)

—

PLEASE NOTE: in the next page, you will make a decision with a different partner

—

(page break)

2. Supplementary References

1. Smith, V. L. Experimental economics: Induced value theory. *Amer. Econ. Rev.* **66**, 274-279 (1976).

2. Balliet, D., Wu, J., & De Dreu, C.K.W. Ingroup favoritism in cooperation: A meta-analysis. *Psychol. Bull.* **140**, 1556–1581 (2014).

3. Ariely, D., Bracha, A. & Meier, S. Doing good or doing well? Image motivation and monetary incentives in behaving prosocially. *Am. Econ. Rev.* **99**, 544–555 (2009).

4. Milinski, M., Semmann, D., Krambeck, H.J. & Marotzke, J. Stabilizing the Earth's climate is not a losing game: Supporting evidence from public goods experiments. *P. Natl. Acad. Sci.* **103**, 3994–3998 (2006).

5. Inglehart, R. & Baker, W. E. 2000 Modernization, cultural change, and the persistence of traditional values. Am. Sociol. Rev. 65, 19–51.

6. Gächter, S., Herrmann, B., & Thöni, C. (2010). Culture and cooperation. Philosophical Transactions of the Royal Society B: Biological Sciences, 365(1553), 2651-2661.

7. Hofstede, G. 2001 Culture's consequences: comparing values, behaviors, institutions, and organizations across nations. Thousand Oaks, CA: Sage

8. Murray, D. R., & Schaller, M.. Historical prevalence of infectious diseases within 230 geopolitical regions: A tool for investigating origins of culture. *J. Cross. Cult. Psychol*. **41**, 99–108 (2010).

9. Schulz, J., Bahrami-Rad, D., Beauchamp, J. & Henrich, J. The Origins of WEIRD Psychology. Working Paper (2018).

10. Falk, A., Becker, A., Dohmen, T., Enke, B., Huffman, D., & Sunde, U. Global evidence on economic preferences. *Q. J. Econ.* **133**, 1645–1692 (2018).

11. Schwartz, S. H., & Boehnke, K. Evaluating the structure of human values with confirmatory factor analysis. *J Res Pers* **38**, 230–255 (2004).

12. Uz, I. (2015). The index of cultural tightness and looseness among 68 countries. *J. Cross. Cult. Psychol*. **46** 319–335.

13. Muthukrishna, M., Bell, A. V., Henrich, J., Curtin, C. M., Gedranovich, A., McInerney, J., & Thue, B. Beyond western, educated, industrial, rich, and democratic (WEIRD) psychology: measuring and mapping scales of cultural and psychological distance. *Psychol. Sci.* **31**, 678–701 (2020).
