# Supplementary material for: National parochialism is ubiquitous across 42 nations around the world
Source: Nat Commun. 2021 Jul 22;12:4456. doi: 10.1038/s41467-021-24787-1 (PMC8298626; doi:10.1038/s41467-021-24787-1)
Supplement: Supplementary file 3 — Reporting Summary [file 41467_2021_24787_MOESM3_ESM.pdf]

nature research

Corresponding author(s): Angelo Romano

Last updated by author(s): Apr 8, 2021

Reporting Summary

Nature Research wishes to improve the reproducibility of the work that we publish. This form provides structure for consistency and transparency in reporting. For further information on Nature Research policies, see our Editorial Policies and the Editorial Policy Checklist.

Statistics

For all statistical analyses, confirm that the following items are present in the figure legend, table legend, main text, or Methods section.

n/a	Confirmed	
☐	☒	The exact sample size (n) for each experimental group/condition, given as a discrete number and unit of measurement
☐	☒	A statement on whether measurements were taken from distinct samples or whether the same sample was measured repeatedly
☐	☒	The statistical test(s) used AND whether they are one- or two-sided<br>*Only common tests should be described solely by name; describe more complex techniques in the Methods section.*
☐	☒	A description of all covariates tested
☒	☐	A description of any assumptions or corrections, such as tests of normality and adjustment for multiple comparisons
☐	☒	A full description of the statistical parameters including central tendency (e.g. means) or other basic estimates (e.g. regression coefficient) AND variation (e.g. standard deviation) or associated estimates of uncertainty (e.g. confidence intervals)
☐	☒	For null hypothesis testing, the test statistic (e.g. F, t, r) with confidence intervals, effect sizes, degrees of freedom and P value noted<br>*Give P values as exact values whenever suitable.*
☒	☐	For Bayesian analysis, information on the choice of priors and Markov chain Monte Carlo settings
☐	☒	For hierarchical and complex designs, identification of the appropriate level for tests and full reporting of outcomes
☐	☒	Estimates of effect sizes (e.g. Cohen's d, Pearson's r), indicating how they were calculated

Our web collection on statistics for biologists contains articles on many of the points above.

Software and code

Policy information about availability of computer code

Data collection	Individual responses were collected using the Qualtrics software.
Data analysis	Data were analyzed using the software R 4.0.5, the lme4 package version 1.1-26, and the metafor package 2.4-0.

For manuscripts utilizing custom algorithms or software that are central to the research but not yet described in published literature, software must be made available to editors and reviewers. We strongly encourage code deposition in a community repository (e.g. GitHub). See the Nature Research guidelines for submitting code & software for further information.

Data

Policy information about availability of data

All manuscripts must include a data availability statement. This statement should provide the following information, where applicable:

- Accession codes, unique identifiers, or web links for publicly available datasets
- A list of figures that have associated raw data
- A description of any restrictions on data availability

Data are accessible here: https://osf.io/68wds/

Field-specific reporting

Behavioural & social sciences study design

All studies must disclose on these points even when the disclosure is negative.

Study description	In this cross-cultural experiment conducted across 42 nations the goal was to understand the existence, ubiquity, and variation of national parochialism across nations. National parochialism can be defined as the tendency to be more cooperative with people belonging to the same nation, compared to people of different nationality or unknown people. To accomplish this goal, in this online experiment participants were asked to make cooperation decisions in a prisoner's dilemma with participants from ingroup, outgroup, and unidentified partners.
Research sample	To improve the generalizability of the results, we decided to recruit representative samples across 42 nations. In each nation, the sample was stratified by age, gender, and income. The average age across nations was 37.40 years old (standard deviation across nations SD = 12.52). The nation with the lowest mean age was Egypt (mean age = 29.44, SD = 8.50), whereas the nation with the highest mean age was Japan (mean age = 48.06, SD = 11.82). Overall, 52.77 % of participants identified as female. The lowest percentage of participants identified as female was in Pakistan (24.48%), whereas the nation with the highest rate of participants that identified as female was Brazil (61.66%).
Sampling strategy	Sample is stratified by age, gender, and income. Our goal was to detect discrimination between ingroup and outgroup members/strangers. A recent meta-analyses found an effect size of d = 0.27 for the within-subjects difference between people's willingness to cooperate with an ingroup member, compared to an outgroup member. An a priori-power analysis suggested that to detect this effect size at statistical power (1-β) = 0.95 and α = 0.05 requires a sample size of 150 people per country. A sensitivity power analyses that consider a sample size of 400 participants and a 95% statistical power and 5% of probability error, revealed that we could detect even very small effect sizes of discrimination (d = 0.16).
Data collection	People were recruited by a panel agency that was not aware of the experimental hypotheses. Data were recorded through the Qualtrics software. Data were collected in a within-subjects design (participants made choices across all experimental treatments). Therefore, the researcher could not influence the results knowing the hypotheses and/or the experimental conditions in advance.
Timing	Data were collected between December 2018 and January 2019. There was no gap in the collection period.
Data exclusions	We had an agreement with the panel agency to not include participants who failed the attention check. In the SI, we report a robustness check including these participants and replicate the results reported in the manuscript.
Non-participation	No participants dropped out or declined participation.
Randomization	We implemented a balanced within-subjects design, hence there was no random allocation to between-subjects treatments. Yet, each experimental treatment was presented in random order to control for order effects. Moreover, in the statistical analyses, we control for subjects by including participant ID as a random factor in all main models (to take into account that some choices may come from one subjects). Moreover, we include age and gender as covariates.

Reporting for specific materials, systems and methods

We require information from authors about some types of materials, experimental systems and methods used in many studies. Here, indicate whether each material, system or method listed is relevant to your study. If you are not sure if a list item applies to your research, read the appropriate section before selecting a response.

Materials & experimental systems

n/a	Involved in the study	
☒	☐	Antibodies
☒	☐	Eukaryotic cell lines
☒	☐	Palaeontology and archaeology
☒	☐	Animals and other organisms
☐	☒	Human research participants
☒	☐	Clinical data
☒	☐	Dual use research of concern

Methods

n/a	Involved in the study	
☒	☐	ChIP-seq
☒	☐	Flow cytometry
☒	☐	MRI-based neuroimaging

Human research participants

Policy information about studies involving human research participants

Population characteristics	See the section "research sample" above.
Recruitment	Participants were recruited online through an external panel agency. Participants were stratified by age, gender, and income. Participants who failed the attention check were excluded. We take this potential self-selection into account by running the a robustness check model with the whole sample and found consistent results.
Ethics oversight	The research was approved by the Massey University Human Ethics Committee, application number: 4000019960 and by the board for Ethical Questions in Science of the University of Innsbruck, application number 37/2018.

Note that full information on the approval of the study protocol must also be provided in the manuscript.
